# ”I Feel Like a Stranger in the Community:” Social Isolation of Older African Immigrants

**DOI:** 10.1093/geroni/igaf122.1625

**Published:** 2025-12-31

**Authors:** Dolapo Adeniji, Onome Osokpo, Gifty Ashirifi, Margaret Adamek

**Affiliations:** Indiana State University, United States; University of Illinois Chicago, Chicago, Illinois, United States; Central State University, Wilberforce, Ohio, United States; Indiana University Indianapolis, Indianapolis, Indiana, United States

## Abstract

Social isolation—limited interactions, relationships, and engagements with others—negatively impacts health outcomes, particularly among older adults, leading to an increased risk of anxiety and depression. Yet little is known about social isolation amongst older African immigrants, a population at increased risk for social isolation. Using a qualitative narrative design, we explored the social isolation experiences of a purposive sample of African immigrants aged ≥60 years recruited across the US (N = 11). In-depth video/phone, audiotaped interviews were completed using a semi-structured guide. Data were analyzed using an inductive thematic approach. The sample was 55% female, 63–79 years old, mostly Nigerian (n = 7), with no primary/secondary education (n = 4), widowed (n = 4), and spouse not in the US (n = 4). All lived with family, and most (n = 10) have lived in the US for < 10 years. We identified two major themes: 1) minimal social engagement outside the family, with participants feeling like strangers in their communities; and 2) barriers to social engagement outside the home, including security concerns, a lack of individuals to socialize with, language barriers and cultural differences, and transportation challenges. Findings indicate that feeling like a stranger, lacking social connections, cultural differences, and lack of transportation contribute to feelings of isolation amongst older African immigrants. African immigrant communities should be educated about the challenges faced by their older adults. Likewise, practitioners and policymakers should prioritize interventions that encourage meaningful engagement of older African immigrants to improve their health and quality of life.

